# Modeling of starter cultures growth for improved Thai sausage fermentation and cost estimating for sausage preparation and transportation

**DOI:** 10.1002/fsn3.708

**Published:** 2018-06-28

**Authors:** Wiramsri Sriphochanart, Wanwisa Skolpap

**Affiliations:** ^1^ Division of Industrial Fermentation Technology Faculty of Agro‐Industry King Mongkut's Institute of Technology Ladkrabang Bangkok Thailand; ^2^ Department of Chemical Engineering School of Engineering Thammasat University Pathumtani Thailand

**Keywords:** flavor, lactic acid bacteria, mathematical modeling, sausage preparation and transportation cost, starter culture

## Abstract

The purpose of this study was to improve Thai fermented sausage flavor by adding starter cultures (i.e., *Pediococcus pentosaceus*,* Pediococcus acidilactici*,* Weissella cibaria*,* Lactobacillus plantarum*,* Lactobacillus pentosus*, and *Lactobacillus sakei*) as compared with naturally fermented sausage. The predictive mathematical models for growth of *P. acidilactici* and natural lactic acid bacteria (LAB) in Thai fermented sausage were developed to obtain specific prepared sausage quality. Furthermore, comparisons of sausage preparation and transportation cost between nonrefrigerated and refrigerated trucks were studied. The concentration of 3‐methyl‐butanoic acid synthesized from LAB inoculated sausage was higher than in the control sample which contributed to the flavor forming. Moreover, the proposed unstructured kinetic models of Thai fermented sausage substrates and products describing the consumption of total protein and glucose, and the production of nonprotein nitrogen responsible for flavor enhancer, lactic acid and formic acid concentration were successfully fitted with two selected experimental data sets of the in situ fermentation of Thai fermented sausage. Finally, the transportation of inoculated sausages in a nonrefrigerated truck by combining fermentation process and transportation was more cost efficient for delivering sausages in a long distance.

## 1 INTRODUCTION

Thai fermented sausage is a mixture of minced pork, fat, cooked rice, garlic, salt, pepper, and soy sauce (Sriphochanart & Skolpap, 2011). The sausage mixture is naturally contaminated by different microorganisms that originated from the raw meat and the environment (Leroy & De Vuyst, 2004). The improvement of fermented sausages is based on the use of starter cultures, particularly lactic acid bacteria (LAB). Microorganisms belonging to the lactobacillus group, that is, *Lactobacillus plantarum*,* Lactobacillus sakei*,* Lactobacillus curvatus*, and *Lactobacillus pentosus* have been employed in meat fermentation (Visessanguan et al., 2006). They initiate rapid acidification of raw meat through the production of organic acids, mainly lactic acid (Demeyer et al., 2000). Acidification at pH value below 5.0 is the basis for the safety as well as quality properties of the end products. In addition, LAB may be added to fermented sausage mixture to enhance flavor formation (Larrouture, Ardillon, Pépin, & Montel, 2000; Leroy, Verluyten, & De Vuyst, 2006).

The flavor of fermented sausages has been widely studied in recent years. These studies have focused on the mechanism involving flavor formation (Herranz, de la Hoz, Hierro, Fernández, & Ordóñez, 2005). Flavor is developed from a complex combination of several volatiles (aldehydes, alcohols, ketones, their acids) and nonvolatile (amines, amino acids, small peptides) compounds. Proteolysis gives rise to the development of the flavor compounds by meat proteins degradation to peptides and free amino acid and the subsequent degradation of amino acids to branched aldehydes and ketones with aroma characteristics (Bruna et al., 2003). Branched‐chain amino acids, that is, leucine, isoleucine, and valine can be converted to flavor enhancing methyl‐branched aldehydes, alcohols, and acids. For example, 2‐ and 3‐methyl‐butanol and, especially, 2‐ and 3‐methyl‐butanal are essential compounds of sausage aroma. Similarly, 2‐, 3‐methyl‐butanoic acid has been reported to be an important contributor to sausage flavor (Søndergaard & Stahnke, 2002). Therefore, this study was undertaken with the hypothesis that the addition of starter cultures to Thai fermented sausage could improve sausage flavor.

During the fermentation of fermented sausage, LAB starter cultures can shift from homo‐ to hetero‐fermentative metabolism under stress conditions, that is, limitation of oxygen, nutrient availability, and high salt concentration, causing production of acetic acid and formic acid (Bobillo & Marshall, 1992). Evidently, the food environment affected the evolution of the microbial populations and the quality attributes. The addition of *Lactobacillus curvatus* 54M16 to a traditional fermented sausage caused hydrolysis of sarcoplasmic protein and reduction of nitrates to nitrites (Casaburi, Martino, Ferranti, Picariello, & Villani, 2016). Subsequently, its use as starter culture could improve quality, safety of the traditional fermented sausage, and flavor acceptance.

Due to the increase in demand of processed products, several researchers have studied alternative technologies for preserving original sensory and nutritional characteristics. Existing preservation technologies are electromagnetic fields, ultrasound technology, ionic radiation, ohmic heating, sterilization by membrane, and high hydrostatic pressure (Pereira & Vicente, 2010). Alternatively, starter culture technology offers consistency of product quality, inhibition of undesirable microbial growth, extension of product shelf life, and transport facilitation without a refrigerated truck. By focusing on LAB, that is, *L. sakei*,* L. plantarum*,* L. pentosus*,* Pediococcus acidilactici,* and *Pediococcus pentosaceus*, are the most commonly used starter cultures for fermented meat products. In the food industry, LAB are added as starter cultures to basic food products, such as milk, meat, vegetables, and cereals, with the purpose to achieve stable and safe end products (Caplice & Fitzgerald, 1999; Zanette, Dalla Santa, & Bersot, 2015). LAB have a potential to produce the antimicrobial lactic acid and hence preserve the food from spoilage bacteria and foodborne pathogen (Leroy & De Vuyst, 2004). Therefore, they can be used to control the fermentation process of fermented sausages by inhibiting the growth of the undesirable microorganisms (Sriphochanart & Skolpap, 2011).

Recently, mathematical models have been used to predict the relation between the food environment and bacterial functionality (Pin & Baranyi, 1998). Particularly, the modeling of functional properties of LAB used as starter cultures in food fermentations has application in design fermentation process. Modeling of growth of pathogens and spoilage organisms in food products was studied such as stochastic modeling and meta‐analysis for *Listeria monocytogenes* growth in fermented sausages (Mataragas, Alessandria, Rantsiou, & Cocolin, 2015) and stepwise regression, Genetic Algorithm‐Artificial Neural network, and Co‐Active Neuro Fuzzy Inference System for prediction of *Pseudomonas aeruginosa* population in Frankfurter sausage (Alghooneh, Alizadeh, Noorbakhsh, & Yazdi, 2015). Only few researches have studied about development of mathematical modeling of starter cultures in food systems such as lactic acid bacteria (LAB) for achieve specific fermentation result.

The two major types of models used in food technology are empirical and fundamental (McDonald & Sun, 1999). Empirical modeling can be applied to fit the experimental data obtained under specific environmental conditions concerning cell growth, sugar metabolism, and the production of functional metabolites. Empirical models are used in food systems to predict the microbial safety or shelf life of products. After these empirical models are obtained, fundamental models can be developed to describe the effect of variation of variables, that is, concentrations of carbon and nitrogen sources on the metabolic responses. Finally, mathematical descriptions of microbial growth must be validated by comparison of calculated values and experimental data. An unstructured model was developed to describe bacterial growth, substrate utilization, and lactic acid production by *L. plantarum* in cucumber juice fermentations (Passos, Fleming, Ollis, Hassan, & Felder, 1993).

In general, the available models have been validated by experimental data derived after growth of various microorganisms in systems of pure cultures (synthetic liquid media or food extracts) under specific conditions. As foods such as meat are complicated systems, the validation of models with data directly from food system in their natural state has been recommended (Whiting & Masana, 1994).

The aim of this work was to investigate the effect of six different external starter cultures such as *P. pentosaceus*,* P. acidilactici*,* Weissella cibaria*,* L. plantarum*,* L. pentosus,* and *L. sakei* on the generation of volatile compounds in Thai fermented sausages. It focused on the degradation of leucine to form 3‐methyl‐butanal, 3‐methyl‐butanol, and 3‐methyl‐butanoic acid. Subsequently, two experiments such as the control batch without inoculation of starter culture and the batch inoculated with starter culture of *P. acidilactici* were selected to verify the proposed predictive unstructured kinetic growth model of LAB in Thai fermented sausages. The kinetic equations of LAB growth, glucose and protein utilizations, lactic acid, formic acid, and nonprotein nitrogen productions were developed. Finally, sausage preparation and transportation costs between nonrefrigerated and refrigerated trucks were analyzed.

## 2 MATERIALS AND METHODS

### 2.1 Microorganism and medium

#### 2.1.1 Microbial strains

The selected LAB strains were *L. plantarum* BCC 4355, *L. pentosus* BCC 5496, and *P. pentosaceus* BCC 4312 obtained from National Center for Genetic Engineering and Biotechnology (BIOTEC), Thailand. *L. sakei* TISTR 890 and *P. acidilactici* TISTR 425 were purchased from Thailand Institute of Scientific and Technological Research. *W. cibaria* JCM 12495 was purchased from Japan Collection of Microorganisms. They were routinely grown in deMan Rogosa Sharpe (MRS) medium at 30°C for 24 hr and then stored at −80°C in fresh medium containing 16% glycerol.

#### 2.1.2 Preparation of starter culture

Pure cultures of selected lactic acid bacteria were inoculated in MRS broth and then incubated at 30°C for 24 hr. Prior to inoculation, the cell concentration was adjusted to give 2 × 10^7 ^CFU/g sausage with sterile deionized water.

### 2.2 Preparation of fermented sausage and sampling

The sausage was prepared from 68% wt ground pork, 25% wt cooked rice, 3% wt garlic, 0.5% wt salt, 0.5% wt black pepper, and 3% wt soy sauce according to the recipe reported in Sriphochanart and Skolpap (2010). During the mixing, the starter culture was added at a dose of 2 × 10^7 ^CFU/g sausage with initial pH ~ 5.87 and then stuffed into air‐dried bovine small intestine. After stuffing into casings and incubation at 30°C, the first sausage samples from each batch were removed and taken as initial value before fermentation (0^th^ hr). The analysis was performed after 0^th^, 12^th^, 24^th^, 36^th^, 48^th^, and 60^th^ hr of fermentation. All seven batches were performed in duplicate with two different experiments.

### 2.3 Predictive modeling

#### 2.3.1 Microbiological analysis

For microbiological analysis a 20‐g sample of fermenting sausage was withdrawn aseptically to a sterile plastic bag containing 180 ml of sterile peptone (0.1% w/v) solution and agitated vigorously for one minute. Appropriate decimal dilutions of the sample solutions were prepared using sterile peptone water, and 0.1 ml of each dilution was spread on selective agar plates in triplicate. The total viable count (TVC) was determined on nutrient agar (NA) and lactic acid bacteria on MRS agar. Bacterial counts were expressed as colony‐forming units per gram of sample (CFU/g). The mean ± standard deviation of the count was calculated using three replicates for each culture time.

#### 2.3.2 Glucose analysis

Glucose concentration during enzymatic hydrolysis of rice was determined using the glucoses HK enzymatic kit (Sigma, New York, NY, USA).

#### 2.3.3 Organic acid analysis

The concentrations of organic acids including lactic acid and formic acid were analyzed by Aminex HPX‐87H (BioRad, Richmond, CA, USA) column of High‐Performance Liquid Chromatography (HPLC) (Agilent 1100 Series, USA), equipped with a DAD detector (Agilent 1200 Series, USA) set at 210 nm (Sriphochanart & Skolpap, 2011).

#### 2.3.4 Extraction of sarcoplasmic and myofibrillar muscle proteins

The protein components in fermented sausage were fractionated according to the method of Visessanguan et al. (2006). Five grams of sausage sample was extracted with 40 ml of the mixture of 15.6 mM Na_2_HPO_4_ and 3.5 mM KH_2_PO_4_, pH 7.5 using a Moulinex homogenizer for 1 min. The precipitate was removed by centrifugation at 4,000 × *g* for 20 min, and the clear supernatant was used for analysis as sarcoplasmic fraction. The retained pellet was further extracted with 40 ml of the mixture of 0.45 M KCl, 15.6 mM Na_2_HPO_4_, 3.5 mM KH_2_PO_4_, pH 7.5 and centrifuged at 4,000 × *g* for 20 min. As a result, the supernatant containing myofibrillar‐enriched fractions was collected and the pellet was discarded.

#### 2.3.5 Extraction of nonprotein nitrogen

A portion of the resultant–sarcoplasmic supernatant was mixed with cold 50% (w/w) trichloroacetic acid (TCA) to a final concentration of 10% (w/w). The precipitate was removed by centrifugation at 4,000 × *g* for 20 min to obtain clear supernatant solution used for analysis as nonprotein nitrogen fraction. The resulting pellet was discarded.

#### 2.3.6 Total protein and nitrogen content analysis

The total protein concentration of sarcoplasmic and myofibrilla proteins was analyzed by the Bradford method (Bradford, 1976). The nitrogen content of sarcoplasmic, myfibrillar protein fractions, and nonprotein fractions was determined by persulfate digestion method (Hach Company, Loveland, USA). After the digestion for converting all forms of nitrogen to nitrate, sodium metabisulfite is added to eliminate halogen oxide interferences. Then nitrate reacts with chromotropic acid under strongly acidic conditions to form a yellow reaction product. Calibration curve was prepared using NH_3_‐N as nitrogen standard at various concentrations of 6.25, 12.5, 25, and 50 g/L. The standards and samples were measured with an absorbance maximum at 410 nm.

#### 2.3.7 Free amino acid analysis

In determination of free amino acids concentration such as leucine and glutamic acid etc., the resultant supernatant–sarcoplasmic fractions prepared by the procedure described in subsection 2.3.4 were extracted and deproteinized with a C18 solid‐phase extraction cartridge. They were derivatized using ortho‐phthalaldehyde (OPA) and 9‐fluorenylmethyl chloroformate (FMOC). One μl of the derivatized samples was then injected onto an amino acid column 200 × 2.1 mm (Agilent Technologies) and guard column (Hypersil ODS, 20 × 2.1 mm, Agilent Technologies) of reverse‐phase high‐performance liquid chromatography (RP‐HPLC) (Agilent 1000 Series, Palo Alto, CA, USA) equipped with a DAD detector (Agilent 1200 Series, Palo Alto, CA, USA) set at 338 nm (Sriphochanart & Skolpap, 2010). The analysis of free amino acid and total amino acid is the same. The sum of free amino acids represents the total free amino acids.

#### 2.3.8 Volatile compound analysis

The volatile compounds such as 3‐methyl‐butanal, 3‐methyl‐butanol and 3‐methyl‐butanoic acid were analyzed by gas chromatography (Shimadzu GC 14‐A, Kyoto, Japan) equipped with a flame ionization detector (FID) using a DB‐624 capillary column (J&W Scientific, 60 m, 0.32 mm i.d., film thickness 1.8 μm). Two milliliters of sausage sample solutions was adsorbed on a 75‐μm carboxen/poly(dimethylsiloxane) (CAR/PDMS) fiber (Supelco, Bellafonte, Pennsylvania, USA) at 40°C for 15 min prior to insertion and were desorbed from the fiber at 220°C for 15 min.

### 2.4 Statistical analysis

Analyses of total protein, nonprotein nitrogen, and total free amino acid for the sausage batches were conducted in triplicate at six different sampling times (0, 12, 24, 36, 48, and 60 hr). The one‐way ANOVA was carried out using the SPSS v.16 software for Windows (SPSS, Chicago, IL, USA) to test significant differences between the control and starter culture fermentation batches at 95% confidence interval (*p *<* *0.05).

### 2.5 Experimental design

In controlling foodborne pathogens, the behavior of LAB starter culture is essentially to be predicted under specific constraints. The kinetic models were developed and then verified with two selected experimental data sets such as the control batch without inoculation of starter culture and the batch inoculated with starter culture of *P. acidilactici*. The measured extracellular metabolites such as concentrations of LAB, total protein, nonprotein nitrogen, lactic acid, formic acid, and glucose were modeled. The assumptions of the proposed kinetic model of Thai fermented sausage were the following: (a) morphology of lactic acid bacteria is doublets (Phalakornkule & Tanasupawat, 2006); thus, the conversion factor of cell concentration of LAB is 2 × 10^−12^ g/CFU (J. M. Scharer, personal communication, 2009); (b) morphological feature of *P. acidilactici* is the mixture of doublets and tetrads (Phalakornkule & Tanasupawat, 2006); thus, the conversion factor of cell concentration is 3 × 10^−12^ g/CFU (J. M. Scharer, personal communication, 2009); (c) pH and lactic acid have minor influence on LAB growth inhibition; (d) total protein is degraded to free amino acid and nonprotein nitrogen; and (e) glucose consumption is mainly responsible for biomass and lactic acid production. The set of differential equations were solved using orthogonal collocation method.

In all experiments of Thai fermented sausage production, the microbial growth prefers a total protein‐limiting to a carbon‐limiting condition. Therefore, the biomass production rate is expressed as the function of total protein concentration as:

LAB concentration (*y*_1_):

(1) $$\frac{dy_{1}}{dt}=\left(1-e^{-k_{3}t}\right)\left(\frac{k_{1}y_{2}}{k_{2}+y_{2}}\right)y_{1}\left(1-\frac{y_{1}}{y_{1,\max}}\right)-k_{12}y_{1}$$

In Equation (1) and all subsequent equations, the term $\frac{dy_{i}}{dt}$ represents the reaction rate for variable *y*_*i*_. The LAB growth model in Equation (1) combines a lag‐phase term, the production of inhibitory end products, the consumption of limited total protein, and cell lysis. The initial lag phase is expressed in terms of exponential decay function. The long lag time of fermentation represented by the term, *k*_3_, causes experimental data acquisition difficult. The lag phase, the adjustment period, is a long‐term consumption of the new nutrient‐rich environment for further exponential cell growth. In perspective of food safety, the lag time estimation is crucial because it involves with prediction of period of pathogen outgrowth in a food product (Schultz & Kishomy, 2013). A Monod model accounts for the effect of the total protein concentration (*y*_2_) on the specific microbial growth rate which is represented by the term, *k*_2_. During exponential growth phase, cell growth reaches its constant rate of maximum specific growth rate, *k*_1_ or μ_max_, and further is mostly inhibited by the *y*_1,max_ term (the maximum cell concentration) and the *k*_12_ term (rate of cell lysis). The value of a monotonic inhibition function, the $1-\frac{y_{1}}{y_{1,\max}}$ term, is approximately between one and zero as suggested by the original logistic model (Fujikawa, Kai, & Morozumi, 2003).

The total protein concentration containing more than 60% of dried weight of Thai fermented sausage was utilized in biomass production and degraded into nonprotein nitrogen. A formation of protein networks and structures accompanied by interaction with other ingredients is essential for the textural, sensory, and nutritional quality of foods (Visessanguan, Benjakul, Riebroy, & Thepkasikul, 2004). As fermentation proceeded, changes in the protein fraction of Thai fermented sausage proteins were illustrated by the decrease in the myofibrillar and sarcoplasmic proteins, accompanied by the increase in nonprotein nitrogen fraction (Sriphochanart & Skolpap, 2010). The nonprotein nitrogen compounds comprise free amino acid, nucleotides, and peptides which are major contributors to flavor forming in fermented sausages (Bruna et al., 2003; Durá, Flores, & Toldrá, 2004). The variations of protein compositions in Thai fermented sausage can be represented by Equations (2) and (3). Equation (2) expresses the degradation of myofibrillar and sarcoplasmic proteins or total protein during the fermentation as follows:

Total protein concentration (*y*_2_):

(2) $$\frac{dy_{2}}{dt}=-cor_{\mathrm{bio}}\left(\frac{N_{\mathrm{bio}}AW_{N}}{MW_{\mathrm{bio}}}\right)\frac{dy_{1}}{dt}+cor_{\mathrm{NPN}}\left(\frac{N_{\mathrm{NPN}}AW_{N}}{MW_{\mathrm{NPN}}}\right)\frac{dy_{3}}{dt}-cor_{X}$$

The *cor*_bio_ term expresses estimating uncertainty of total protein in biomass empirical formula. *cor*_NPN_ accounts for analytical measurement discrepancy of total protein degradation.

The proteolysis of muscle myofibrilla proteins causes higher accumulation of soluble nonprotein nitrogen compounds and formation of volatile compounds served as flavor enhancers (Benito, Núñez, Córdoba, Martin, & Córdoba, 2005). The production rate of nonprotein nitrogen was modeled by the Luedeking–Piret equation comprising growth‐associated and nongrowth‐associated terms. The model is defined by Equation (3):

Nonprotein nitrogen concentration (*y*_3_):

(3) $$\frac{dy_{3}}{dt}=-k_{4}\frac{dy_{2}}{dt}+k_{5}y_{2}$$

Lactic acid is derived from pyruvate with the aid of lactate dehydrogenase. Lactic acid effectively inhibits pathogenic growth (Marc, Valik, & Med′vedová, 2009). Commonly, lactic acid production is predicted by growth‐associated model (Poschet, Vereecken, Geeraerd, Nicolï, & Van Impe, 2005). Lactic acid synthesis was modeled by the Luedeking–Piret equation with lactic acid inhibition term and can be expressed as follows:

Lactic acid concentration (*y*_4_):

(4) $$\frac{dy_{4}}{dt}=k_{6}\frac{dy_{1}}{dt}+k_{7}\left(1-\frac{y_{4}}{y_{4,\max}}\right)$$

The variable term, *k*_6_, represents a quantity of lactic acid synthesized by cell division. As aforementioned, the pathogen growth inhibition may take place before its maximum microbial concentration, *y*_1,max_, is reached (Marc et al., 2009).

The presence of organic acids such as acetic acid and formic acid can be attributed to a metabolic shift from homo‐ to hetero‐fermentation of selected LAB starter cultures under stress environmental conditions such as limitation of oxygen, nutrient and salt concentrations, and low pH level (Stiles & Holzafel, 1997). In a similar vein, the model for formic acid formation as a byproduct‐organic acid during hetero‐fermentative metabolism of LAB can be written as follows:

Formic acid concentration (*y*_5_):

(5) $$\frac{dy_{5}}{dt}=k_{8}\frac{dy_{1}}{dt}+k_{9}\left(1-\frac{y_{5}}{y_{5,\max}}\right)$$

Equation (5) describes growth‐associated formic acid formation accounting inhibitory effect of the formation of formic acid at high concentrations.

Pork is a major source of protein, but not a good source of carbohydrate. Thus, the sausage mixture was supplemented with rice served as additional carbon source in fermentation for energy production. The metabolic pathways of carbohydrate are oxidative and glycolytic pathways. Postmortem glycolysis in muscle transforms glycogen, a multibranched polysaccharide of glucose and a main energy storage, into lactic acid (Pereira da costa & Conte‐Junior, 2015). Subsequently, the lactic acid generated is oxidatively converted back to pyruvate via the tricarboxylic acid cycle. The predominant acid in muscle tissue is the lactic acid generated via glycolysis. Glucose uptake was presumably contributed for cell growth and lactic acid synthesis as represented by *k*_10_ and *k*_11_ terms in Equation (6)_,_ respectively. The glucose utilization was inhibited by the maximum concentration of lactic acid produced. Therefore, the glucose model equation can be expressed as:

Glucose concentration (*y*_6_):

(6) $$\frac{dy_{6}}{dt}=-k_{10}\frac{dy_{1}}{dt}-k_{11}\left(1-\frac{y_{4}}{y_{4,\max}}\right)$$

## 3 RESULTS AND DISCUSSION

### 3.1 Proteolysis

The effect of starter cultures on total protein nitrogen, nonprotein nitrogen, and total amino acid in Thai fermented sausages is shown in Table 1.

**Table 1:** Effect of LAB starter cultures on total protein nitrogen, nonprotein nitrogen, and total amino acid in Thai fermented sausages

Sausage samples	Total protein nitrogen (g/100 g sausage)		Nonprotein nitrogen (g/100 g sausage)		Total amino acid (g/100 g sausage)	
	0 hr	60 hr	0 hr	60 hr	0 hr	60 hr
Control	0.563 ± 0.023	0.204 ± 0.011^A^	0.109 ± 0.006	0.192 ± 0.008^A^	0.260 ± 0.030	0.409 ± 0.011^A^
*P. pentosaceus*	0.486 ± 0.019	0.109 ± 0.039^D^	0.109 ± 0.004	0.227 ± 0.004^B^	0.257 ± 0.027	0.478 ± 0.039^B^
*P. acidilactici*	0.513 ± 0.017	0.118 ± 0.021^D^	0.127 ± 0.004	0.254 ± 0.004^D^	0.365 ± 0.026	0.707 ± 0.021^EF^
*W. cibaria*	0.452 ± 0.013	0.115 ± 0.031^D^	0.118 ± 0.004	0.256 ± 0.005^D^	0.383 ± 0.029	0.619 ± 0.031^C^
*L. pentosus*	0.516 ± 0.011	0.069 ± 0.031^C^	0.103 ± 0.006	0.239 ± 0.009^B^	0.343 ± 0.020	0.666 ± 0.031^CF^
*L. plantarum*	0.495 ± 0.010	0.025 ± 0.036^B^	0.118 ± 0.007	0.262 ± 0.008^D^	0.395 ± 0.034	0.722 ± 0.036^E^
*L. sakei*	0.539 ± 0.012	0.060 ± 0.028^C^	0.101 ± 0.004	0.275 ± 0.007^C^	0.344 ± 0.019	0.558 ± 0.028^D^

^A–F^Means within the same column with different superscript letters are different (*p *<* *0.05).

The concentration of total protein nitrogen decreased whereas the concentrations of nonprotein nitrogen and total amino acid increased throughout the fermentation. This was attributed to the initial breakdown of oligopeptides and small peptides containing in dry fermented sausage by endogenous muscle proteinases resulting in the increase of free amino acids and additional peptides due to microbial proteinases (Johansson, Berdague, Larsson, Tran, & Borch, 1994). At 60 hr of fermentation time, Thai fermented sausages inoculated with LAB showed significant decreases in total protein nitrogen (*p *<* *0.05). Nonprotein nitrogen is an indicator for the degree of proteolysis in fermented sausage. The increase in nonprotein nitrogen was highest in *L. sakei* inoculated sausage and lowest in the control sample. The result was consistent with the finding of Fadda et al. (1999) that exoproteolytic activity of *L. sakei* CECT 4808 produced free amino acids responsible for the process of flavor improvement and/or precursors of other flavor compounds. At the end of fermentation, a concentration of nonprotein nitrogen observed in LAB inoculated sausages was significantly higher than the control (*p *<* *0.05). A greater increase in total free amino acid was also detected in sausages inoculated with LAB compared to the control (Table 1). Hughes et al. (2002) reported similar results. The hydrolysis of meat proteins generates polypeptides that can be further degraded to smaller peptides and free amino acid by various microbial and endogenous muscle enzymes (Durá et al., 2004; Hughes et al., 2002). Demeyer et al. (2000) implicated exopeptidases from *L. sakei* together with muscle aminopeptidase as being responsible for the generation of free amino acid from the N‐amino terminal of muscle proteins and peptides. Free amino acid could be transformed into volatile compounds, resulting in an enhancement of flavor of dry fermented sausage. However, the results of individual free amino acid synthesis during sausage fermentation are difficult to compare with other previous studies due to condition differences such as time, temperature, and microflora of the sausage mixture (Shadi, 2014).

### 3.2 Change in leucine and glutamic acid concentration

**Figure 1:**
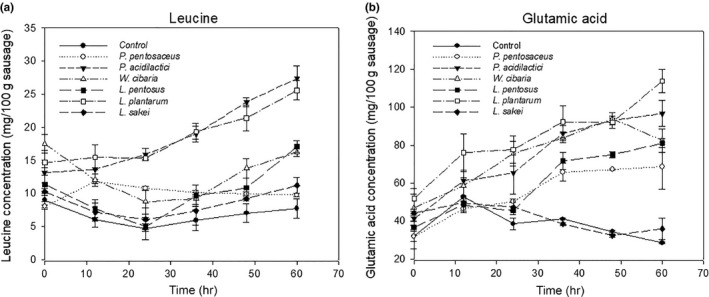
Concentration profiles in Thai fermented sausages with/without starter cultures during fermentation: (a) leucine and (b) glutamic acid

The control sample and sausages inoculated with *W. cibaria*,* L. pentosus,* and *L. sakei* showed a decrease in leucine concentration until 24 hr due to leucine catabolism. Afterward, a slight increase of leucine level was caused by the proteolytic degradation of nonprotein nitrogen (as shown in Figure 1a). In sausages inoculated with *L. plantarum* and *P. acidilactici*, the concentration of leucine remained unchanged while the glutamic acid concentration was significantly increased for 24 hr (as shown in Figure 1b).

**Figure 2:**
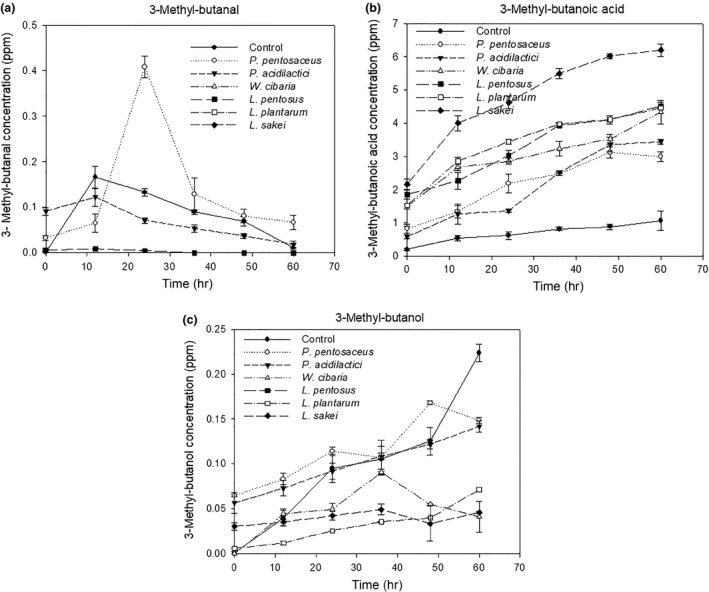
Volatile compound profiles of Thai fermented sausages inoculated with/without LAB starter cultures; (a) 3‐methyl‐butanal, (b) 3‐methyl‐butanoic acid, and (c) 3‐methyl‐butanol

This may be attributed that nitrogen is removed from leucine by transaminase and transferred to α–ketoglutarate to form glutamic acid (Smit, Smit, & Engels, 2005) as leucine is a predominant branched‐chain amino acid during consumption of high‐protein substrate (Kanamori, Ross, & Kondrat, 1998). Thereafter, leucine was progressively increased as the transamination is a reversible reaction depending on which of the reactant is in excess. At the end of fermentation, the concentration of leucine observed in sausages inoculated with LAB starter cultures was higher than in the control. The appearance may be attributed that LAB starter cultures provide more enzymatic conversion of nonprotein nitrogen to peptides and free amino acids especially leucine served as a major precursor of volatile compounds such as 3‐methyl‐butanal, 3‐methyl‐butanoic acid, and 3‐methyl‐butanol via α–ketoisocaproic acid (Smit et al., 2005) under acidic condition (Kanamori et al., 1998). The profiles of volatile compounds of prepared sausages are shown in Figure 2.

The production of 3‐methyl‐butanal was observed from sausages inoculated with *P. pentosaceus*,* P. acidilactici*,* L. pentosus,* and the control. The concentration of 3‐methyl‐butanal obtained from a batch inoculated with *P. pentosaceus* reached the highest value at 24 hr. As shown in Figure 2a, the concentration of 3‐methyl‐butanal synthesized from *P. acidilactici* and the control batches was increased during the first 12 hr of fermentation and subsequently decreased until the end of fermentation, probably due to its reduction to 3‐methyl‐butanol by alcohol dehydrogenase. Sausage inoculated with *L. pentosus* yielded the lowest concentration of 3‐methyl‐butanal. Thai fermented sausage inoculated with LAB starter cultures showed higher concentrations of 3‐methyl‐butanoic acid than the control (Figure 2b). The same results of the production of 3‐methyl‐butanoic acid derived from leucine catabolism in fermented sausages inoculated with LAB were reported in the previous studies (Demeyer et al., 2000; Larrouture et al., 2000). This may be attributed that the conversion of leucine to 3‐methyl‐butanoic acid is the most energetically favorable pathway to generate one mol of ATP per mol of leucine (Kanamori et al., 1998). The branched alcohol, 3‐methyl‐butanol, derived from the aldehyde, 3‐methyl‐butanal, was observed in sausages inoculated with *P. pentosaceus*,* P. acidilactici*,* W. cibaria*,* L. plantarum, L. sakei,* and control sample (Figure 2c). The concentration of 3‐methyl‐butanol increased throughout the fermentation. At the end of fermentation, control sample showed the highest concentration of 3‐methyl‐butanol. The volatile compounds 3‐methyl‐butanal and 3‐methyl‐butanoic acid, derived from leucine by corresponding ketoacid decarboxylation and oxidative decarboxylation, have a strong effect on sensory quality traits of fermented sausages (Larrouture et al., 2000; Leroy et al., 2006). Evidently, the LAB starter could accelerate the degradation of leucine to 3‐methyl‐butanoic acid rather than to 3‐methyl‐butanal. Moreover, synthesis of volatile compounds derived from Italian fermented sausages was related to types of starter cultures (Montanari et al., 2016).

Thai fermentation sausages inoculated with *P. acidilactici* received the best taste, texture, and overall preference scores (*p *<* *0.05), followed by *L. pentosus, L. plantarum, W. cibaria, P. pentosaceus, L. sakei,* and control (Sriphochanart & Skolpap, 2011). The sensory result is consistent with the study of Herranz et al. (2005) that the better flavor score of sausages inoculated with LAB starter cultures could be related to the higher concentration of volatile compounds derived from leucine.

To predict microbial growth, production of end products and the consumption of sausage nutrients for quality and safety improvement of the fermented sausage, modeling the effect of LAB starter culture growth on proteolysis, and flavor enhancement were further developed.

### 3.3 Modeling results and discussion

#### 3.3.1 Model parameter estimation

The set of differential Equations (1) to (6) was solved simultaneously using orthogonal collocation. Two experimental data sets, that is, control and *P. acidilactici* inoculated experiments, were applied to estimate parameter values and to calibrate the model using the Gibbs parameter sampling approach (Gilks, Richardson, & Spiegelhalter, 1998). To identify set of parameter estimates based on statistical analysis, the sampled parameter vector space was repeatedly generated by a Monte Carlo draw at least 10,000 trials until the acceptance criterion of Gilks et al. (1998) was satisfied.

**Figure 3:**
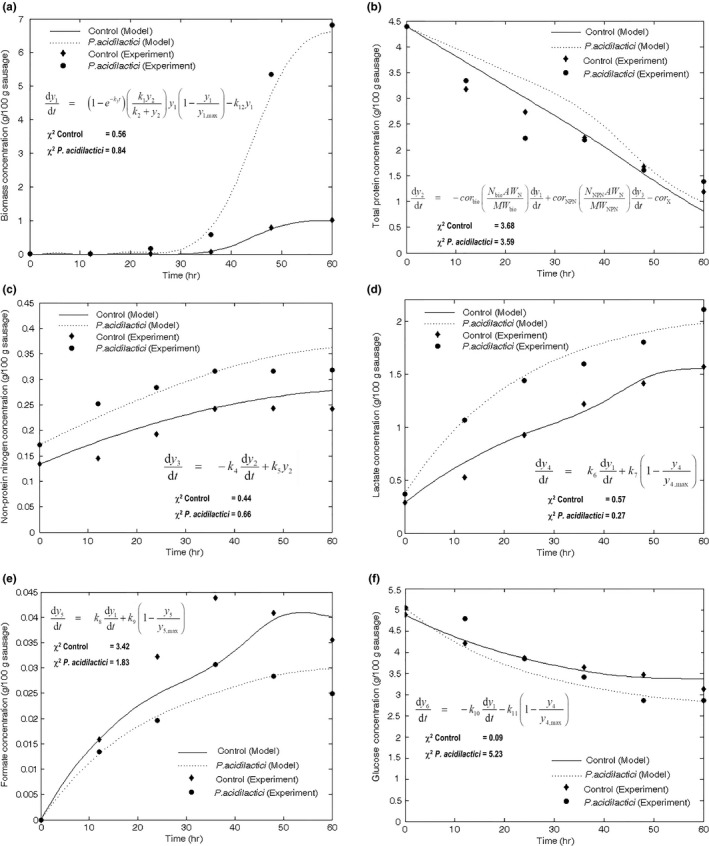
Modeled and experimental results: (a) Biomass concentration (*y*_1_), (b) total protein concentration (*y*_2_), (c) nonprotein nitrogen (*y*_3_), (d) lactic acid concentration (*y*_4_), (e) formic acid concentration (*y*_5_), and (f) glucose concentration (*y*_6_)

The chi‐squared goodness of fit was used to evaluate nonparametrically the statistic discrepancy between observed and predicted measurements under the developed models at 95% confidence level. The results of modeling are illustrated in Figure 3a–f. The model parameter values of control and *P. acidilactici* inoculated experiments are listed in Table 2.

**Table 2:** Estimated values of model parameters

Model parameter	Value for control experiment	Value for *P. acidilactici* inoculated experiment
*k*_1_	0.330	0.244
*k*_2_	0.061	0.088
*k*_3_	0.142	0.141
*k*_4_	3.7 × 10^−5^	4.64 × 10^−4^
*k*_5_	1.517 × 10^−5^	1.275 × 10^−5^
*k*_6_	0.414	1.112 × 10^−3^
*k*_7_	0.064	0.095
*k*_8_	0.013	2.623 × 10^−4^
*k*_9_	1.458 × 10^−3^	1.7498 × 10^−3^
*k*_10_	9.855 × 10^−3^	6.674 × 10^−3^
*k*_11_	0.101	1.280
*k*_12_	1.7 × 10^−3^	1.9 × 10^−3^
*cor*_bio_	1.118	1.094
*cor*_NPN_	0.832	0.723
*cor*_X_	0.025	0.046

The growth model was validated by comparing experimental data of sausage inoculated with *P. acidilactici* and that of sausage without inoculation (control) and their corresponding growth predicted by model Equation (1). The growth curves of natural flora (control) and *P. acidilactici* in sausage fermentation showed long lag phase (Figure 3a). The longer the lag phase, the longer the natural flora or starter culture prevail against the pathogens, which in turn, benefits food safety (Madar et al., 2013). Several available growth models cannot accurately predict the lag time which is used in estimating food shelf life (Madar et al., 2013; McKellar, 1997). As there are a variety of factors affecting the lag parameter, the lag time constant is usually more difficult to predict than the specific growth rate (McKellar, 1997). The predicted results of both experiments showed a good agreement with the observed results using Equation (1) to describe the growth of *P. acidilactici* and natural LAB in Thai fermented sausage as shown in Figure 3a. All of estimated chi‐squared values as shown in Figures 3a–f were within the listed 95% probability limit.

The predicted dissolved total protein concentration was compared with observation as presented in Figure 3b. It illustrates the decrease of total proteins during sausage fermentation. The protein fraction is a precursor of most volatile compounds contributing flavor of fermented meat (Demeyer et al., 2000) and that of amino acids contributing microbial growth (Fadda, Oliver, & Vignolo, 2002). For both experiments, the predicted values of dissolved total protein concentration were higher than observed values. It may be attributed that sarcoplasmic and myofibrillar proteins became denatured or insoluble due to acid‐induced degradation (Visessanguan et al., 2004). It was suggested that an induction term of lactic acid or formic acid formation should be included in Equation (2) to achieve better agreement with observed values. However, the estimated chi‐squared probability of total protein concentration of sausage inoculated with *P. acidilactici* was relatively higher than that of control batch.

The predicted dissolved nonprotein nitrogen concentration showed a good fit with observation as presented in Figure 3c. The estimated chi‐squared probability of nonprotein nitrogen concentration of sausage inoculated with *P. acidilactici* was relatively higher than that of the control batch. The increase of nonprotein nitrogen yielding free amino acid increase (see Table 1) was associated with the proteolytic activity during the sausage fermentation (Shadi, 2014). Consequently, the proteolysis was a main process causing aroma improvement in dry sausage (Johansson et al., 1994).

The modeled results of lactic acid concentration, desirable product, were in good agreement with the observed results as shown in Figure 3d. The estimated chi‐squared probability of lactic acid concentration was small in all experiments.

In contrast to control batch, the modeled results of formic acid concentrations obtained from sausage inoculated with *P. acidilactici* fitted well with the experimental results as shown in Figure 3e. Moreover, the estimated chi‐squared probability of formic acid concentration of control batch was relatively higher than that of sausage inoculated with *P. acidilactici*. The modeling agreement of formic acid and glucose concentrations can be improved by measurement of other metabolites such as acetic acid and ethanol in sausage sample for further study. Consequently, the utilization of glucose model Equation (6) is suggested to include more terms for formation of other organic acids and ethanol.

The calculated chi‐squared probability of glucose uptake in control batch was relatively small, while that of glucose uptake in sausage inoculated with *P. acidilactici* was higher. Therefore, the modeled results showed a good agreement with the observed result (Figure 3f) which was verified by the estimated chi‐squared values within the listed 95% probability limit. Thus, the hypothesis was validated that glucose consumption in sausage fermentation was responsible for LAB growth and lactic acid synthesis.

### 3.4 Cost estimating of sausage preparation and transportation

Not only does the addition of LAB starter cultures facilitate preparation of fermented Thai sausages but also offers more cost‐efficient integrated fermentation process and transportation. Two transportation methods based on annual transporting days of 330, that is, refrigerated and nonrefrigerated 6‐wheel trucks were studied by estimating costs of raw materials, operating, and transportation.

Total relevant transportation cost of sausage in chilled or frozen form and that of sausage without refrigeration are expressed in Equation (7) as follows:

(7) $$\text{TC}=C_{0}+C_{1}t_{\mathrm{dr}}+C_{2}t_{\mathrm{dr}}+C_{3}t_{\mathrm{dr}}$$

Each load of 6‐wheel truck is 4.2 tons of sausages. The detailed cost estimated on the basis of full truckload is illustrated in Table 3. These products have the same raw material cost. The preparation of unfermented sausages can save labor cost in the manufacturer due to simultaneous fermentation and nonrefrigerated transportation; therefore, its labor cost was presumably 20% lower than that of preparing 36‐hr fermented sausages in the processing plant. Fuel costs at speed limit of 60 km/hr were calculated based on diesel consumption rate of refrigerated truck and nonrefrigerated truck were 0.20 and 0.08 USD/km, respectively (Anthony, 2012). A truck initial purchase cost‐based depreciation rate was identical for refrigerated truck and nonrefrigerated truck which was 2,857.14 USD/year. A carrying driver could take two 4‐hr driving period of 30‐min sleeper berth with a maximum of 8‐hr driving limit in 24 hr (Urata, 2010). The driver is paid twice the normal wage rate for overtime worked.

**Table 3:** Formula and data for calculating cost

Total production cost (*C*_0_)	Cost (USD/load)
*C*_0_ = *C*_M_ + *C*_L_ where *C*_M_ = raw material cost of sausage manufacturing (Pitcha ( )meat, n.d.) *C*_L_ = labor cost of production based on minimum wage rate of 9.14 USD per 8‐hr day (Supakijjanusorn, 2017) Assume *C*_L_ required for preparing unfermented sausage is 20% lower than 36‐hr fermented sausage	12,333.58 836.57 for refrigerated truck 708.57 for nonrefrigerated truck
Fuel cost (*C*_1_)	Cost (USD/min)
where Diesel price = 0.72 USD/liter (Energy policy and planning office,n.d.) Fuel consumption rate (Anthony, 2012) = 3.6 min/liter for refrigerated truck = 9.0 min/liter for nonrefrigerated truck *C*_1_ at speed limit of 60 km/hr or 1 km/min	0.20 for refrigerated truck 0.08 for nonrefrigerated truck
Depreciation cost (*C*_2_)	Cost (USD/min)
where Annual depreciation rate at 20% of asset cost (A business guide to Thailand, 2014) Assume asset cost of 14,285.71 USD/truck $C_{2}=\frac{0.2\times 14,285.71}{t_{\mathrm{tr}}}$ *t*_tr_ = annual transporting time (min/year) = 8 hr × 330 day/year × 60 min/hr	0.009
Truck driver wage (*C*_3_)	Cost (USD/min)
where During 9‐hr driving period including two 30‐min sleeper‐berth break: *C*_3_ = 14.29 USD (Thailand average salaries and expenditures, n.d.) After the 9‐hr driving period at twice the normal wage rate of pay: *C*_3_ = 2 × 14.29 USD	0.027 0.054

**Figure 4:**
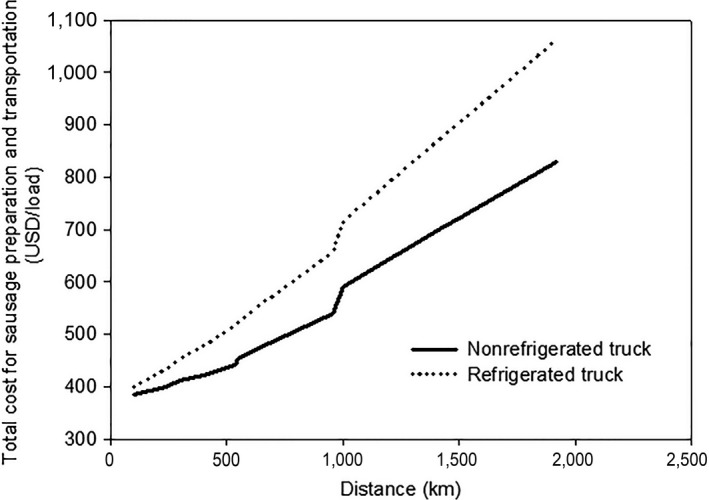
Total preparation and transportation cost of sausage by nonrefrigerated and refrigerated trucks

Comparative total costs of preparation and transportation of frozen and unfrozen sausages relating with delivering distance are represented by monotonic increasing cost function due to the overtime pay for drivers as shown in Figure 4. As the delivering distance increased, the total cost of nonrefrigerated truck was significantly lower than that of refrigerated truck due to less fuel consumption rate of nonrefrigerated truck.

## 4 CONCLUSION

LAB starter cultures used in Thai fermented sausages cause improvement of acidification by higher production of organic acids, mainly lactic acid. The effect of proteolytic activity of LAB caused the decrease in the total protein nitrogen fraction, the increase of nonprotein nitrogen fraction, and total free amino acid throughout the fermentation period. Moreover, LAB had influence on flavor improvement of the product. The concentration of volatile compound derived from leucine, 3‐methyl‐butanic acid, was higher in LAB inoculated samples than in control samples and was the principal flavor‐enhancing compound. The proposed mathematical model of the total protein‐limiting LAB growth in Thai fermented sausage accurately predicted the long lag phase resulting in estimating shelf life of the product. To improve the fitness of total protein model, it was suggested to supplement an induction term of lactic acid or formic acid formation. The Luedeking–Piret model was suitable to predict formation of nonprotein nitrogen contributing for flavor improvement, lactic acid, and formic acid. The transportation of inoculated sausages without refrigeration required was more economical for delivering sausages in a long distance.
